# Polymorphic Forms of Valinomycin Investigated by NMR Crystallography

**DOI:** 10.3390/ijms21144907

**Published:** 2020-07-11

**Authors:** Jiří Czernek, Jiří Brus

**Affiliations:** Institute of Macromolecular Chemistry, Czech Academy of Sciences, Heyrovsky Square #2, 16206 Prague, Czech Republic; brus@imc.cas.cz

**Keywords:** valinomycin, antiviral, 2019-nCoV, solid-state NMR, NMR crystallography, DFT, GIPAW

## Abstract

A dodecadepsipeptide valinomycin (VLM) has been most recently reported to be a potential anti-coronavirus drug that could be efficiently produced on a large scale. It is thus of importance to study solid-phase forms of VLM in order to be able to ensure its polymorphic purity in drug formulations. The previously available solid-state NMR (SSNMR) data are combined with the plane-wave DFT computations in the NMR crystallography framework. Structural/spectroscopical predictions (the PBE functional/GIPAW method) are obtained to characterize four polymorphs of VLM. Interactions which confer a conformational stability to VLM molecules in these crystalline forms are described in detail. The way how various structural factors affect the values of SSNMR parameters is thoroughly analyzed, and several SSNMR markers of the respective VLM polymorphs are identified. The markers are connected to hydrogen bonding effects upon the corresponding (^13^C/^15^N/^1^H) isotropic chemical shifts of (C_O_, N_amid_, H_amid_, H_α_) VLM backbone nuclei. These results are expected to be crucial for polymorph control of VLM and in probing its interactions in dosage forms.

## 1. Introduction

Intense efforts are ongoing to identify treatments of the 2019 novel coronavirus (2019-nCoV) [1]. For an obvious reason, it is a very quickly evolving field that was already reviewed [2,3,4]. Most recently, a press release [5] appeared that outlined the potential of valinomycin (VLM; a cyclodepsipeptide [6] described below) in a treatment of the 2019-nCoV. This potential is assumed on the basis of the most recent report of an efficient preparation of VLM [7] and on its well-known antiviral activity [8]. We thus surmise that VLM may indeed become a part of some 2019-nCoV drug formulation(s) and investigate crystalline phases of VLM. Their description furthers the understanding of the polymorphism of VLM (the ability of a solid VLM to form various crystal modifications). The polymorphism of VLM is expected to be extensive (see the recent study of a related problem of complexation-induced structural changes in VLM [9], and references cited therein) and important for the design and manufacturing of a drug formulation. Due to a great significance of the polymorphism for pharmaceutical industry, there is a number of methods dealing with various aspects of this problem [10]. One of the approaches to the description of polymorphic forms of drug compounds is an application of the NMR crystallography [11] in its variant, most recently reviewed in reference [12], which combines the solid-state NMR (SSNMR) and computational methods together with an input from the X-ray diffraction (XRD) measurements. This NMR crystallography variant is employed in the present study. It applies the plane-wave density-functional theory (PW DFT)-based calculations of the structural and spectral parameters [13] in order to describe several crystal modifications of VLM. Two of those solid phase structures, namely, the triclinic and monoclinic forms, both featuring so called “asymmetric bracelet” conformation of the backbone (see references [14,15], respectively), were previously characterized by the SSNMR [16]. They are used for a comparison of the PW DFT and experimental results. On the basis of this comparison, two additional crystal geometries of VLM are investigated. The first one is called “symmetrical”, as it is predicted from the XRD structure of VLM—K^+^ complex [17], whose shape resembles a circular bracelet, after a removal of the counterions. The second predicted geometry exhibits the “propeller” structural motif (bearing resemblance to a three-blade propeller), and is obtained from the XRD structure of dimethyl sulfoxide solvate of VLM [18]. For all the aforementioned structures, their intermolecular interactions; the key internal coordinates including parameters of hydrogen bonds; and selected SSNMR data are discussed. These results should be useful also in analyses of other pharmaceutically active cyclic peptides like cyclosporins [19,20].

## 2. Results

### 2.1. General Considerations

**Figure 1:**
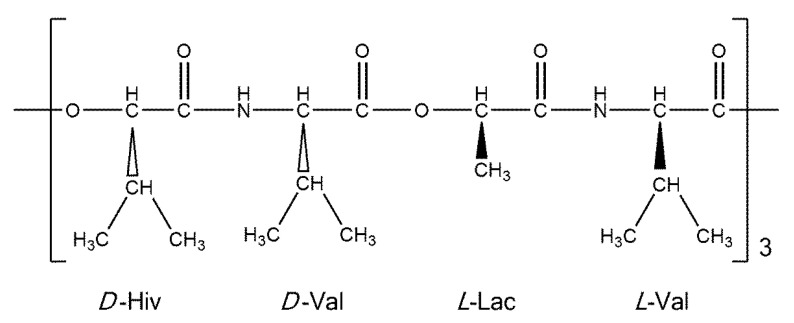
A schematic representation of valinomycin.

Cyclo(*D*-α-hydroxyisovaleryl-*D*-valyl-*L*-lactoyl-*L*-valyl-*D*-α-hydroxyisovaleryl-*D*-valyl-*L*-lactoyl-*L*-valyl-*D*-α-hydroxyisovaleryl-*D*-valyl-*L*-lactoyl-*L*-valyl) (valinomycin, VLM; CAS number 2001-95-8) is a cyclic dodecadepsipeptide with the ring closed at an ester linkage (see Figure 1). It contains three repetitions of (*D*-Hiv–*D*-Val–*L*-Lac–*L*-Val) segment in a macrocycle. Due to an alternation of amide and ester linkages throughout the VLM backbone, we define the backbone dihedral angles *φ* and *ψ* as follows. A quintuple of consecutive atoms {*X*, C_O_, C_α_, *Y*, *Z*} is used: *X* denotes either amidic nitrogen (N_amid_) or ester oxygen of the preceding residue in the twelve-membered ring; C_O_ and C_α_ are the carbonyl and alpha carbons, respectively, of the residue in question; *Y* denotes either N_amid_ or ester oxygen of the residue in question; and *Z* denotes the carbonyl carbon of the subsequent residue (the ordering and atom numbers are specified in the Supporting Materials Tables S1–S4). Then *φ* is the *X*, C_O_, C_α_, *Y* torsion angle, while *ψ* is the C_O_, C_α_, *Y*, *Z* torsion angle. The [*φ*, *ψ*] values are shown below for all the investigated structures.

Importantly, none of the PW DFT optimized periodic structures (see Materials and Methods) of VLM contains any typical O…H–O or O…H–N intermolecular hydrogen bonds. For those structures, the dimerization energy, *ΔE*, of the closest neighboring structural units in a given VLM crystal was obtained by means of the RI-MP2/TZVP supermolecular interaction energy calculations which are described in Section 4. These complexation energies were found to be small for all four polymorphs: *ΔE* values amount to −22.7, −26.5, −19.4, and −36.1 kJ/mol for the dimers taken from the triclinic, monoclinic, “symmetric” and “propeller” crystal structures, respectively. They are thus comparable to the interaction energy of, for instance, an *N*-methylacetamide–dimethylformamide (NMA–DMF) dimer (*ΔE* = −26.9 kJ/mol for the PBE/aug-cc-pVQZ minimum featuring a typical hydrogen bond between the amide group of NMA and the formyl oxygen of DMF). An accuracy of the *ΔE* data is discussed in Section 3.

The NMR isotropic chemical shielding as obtained from the GIPAW calculations (see Materials and Methods) was converted to an estimate of the isotropic chemical shift according to references [21] (^13^C and ^1^H) and [22] (^15^N). These estimates are shown in Table 1, Table 2, Table 3 and Table 4 and discussed below, while the MAGRES files are obtainable from the corresponding author upon request. The tables also describe an arrangement of hydrogen bonds in the respective structures by designating, wherever applicable, a residue as the acceptor (“a”) and/or as the donor (“d”) involved in hydrogen bonding. The actual values of internal coordinates can be inferred from the coordinates which are provided, in PDB format files, in the Supporting Materials.

### 2.2. The Triclinic Polymorph

**Figure 2:**
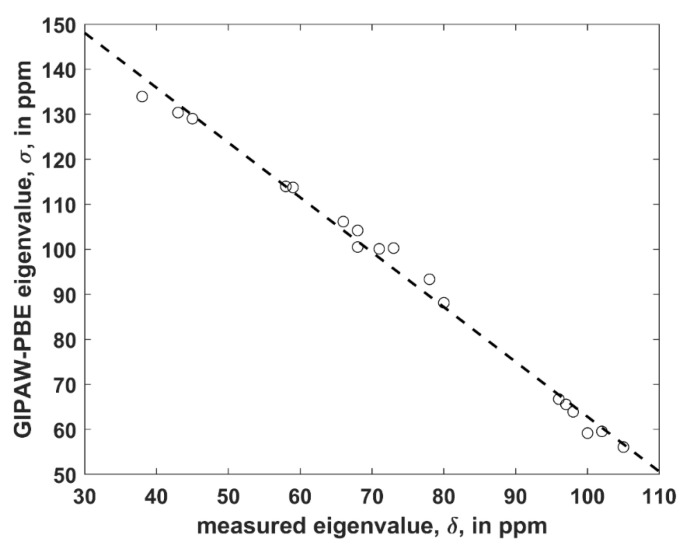
The linear regression model of the ^13^C SSNMR data described in the text (the dashed line is σ = −1.218 × δ + 184.6 ppm).

The structure was considered that is uncomplexed and crystallizes in *P*1 space group with two crystallographically distinct VLM molecules (further referred to as “A” and “B”; an identification of either unit in the Supporting Materials file TRICLINIC.PDB is possible through the numbering provided in Table S1). It was employed to establish a level of agreement between the PW PBE-predicted values of the principal elements of the ^13^C chemical shielding tensors and their measured counterparts, namely, the principal elements of the ^13^C chemical shift tensors. Only α carbons of *D*-Hyv and *L*-Lac sites were considered, as all six corresponding peaks were identified in the ^13^C SSNMR spectrum (the region of carbonyl carbons was not fully resolved) [16]. However, it should be noted that the explicit assignment of those peaks was not obtained by Kameda et al. [16] and hence it was taken from the calculations. It should also be noted that averaged values for molecules “A” and “B” were provided by the experiment [16]. Consequently, the computed results for molecules “A” and “B” were also averaged (raw data before averaging are shown in Table S5). Considering known limitations of PW DFT predictions of the principal components of the ^13^C chemical shielding tensors [23] and uncertainties of SSNMR measurements, the agreement between theory and experiment can be considered to be very good. Namely, a simple linear regression model (σ = *a* × δ + *b*, where σ and δ is the shorthand notation for properly ordered values of the principal elements of the ^13^C chemical shielding and chemical shift tensors, respectively, *a* is the slope, and *b* is the intercept; see Figure 2 has an adjusted *R*^2^ value of 0.9915, standard deviation 2.3 of ppm, and a maximum deviation of 4.6 ppm. This level of accuracy is comparable to ca. 2 ppm error of an extraction of the eigenvalues by fitting of the ^13^C SSNMR spectrum [16].

The triclinic polymorph features a remarkable pattern of six hydrogen bonds (see Table 1). While this pattern is well-known [14], it is investigated by the PW PBE calculations and described in detail in order to facilitate a search for the SSNMR markers identifying the polymorphs of VLM. Four of the hydrogen bonds are of the 1 → 4 type. Using “a → d” notation to specify an acceptor—donor pair, these contacts are: *D*-Hyv_1_ → *L*-Val_4_, *L*-Lac_11_ → *D*-Val_2_, *D*-Hyv_5_ → *L*-Val_8_, and *L*-Lac_7_ → *D*-Val_10_. Two hydrogen bonds are of the 1 → 5 type, namely, *D*-Val_2_ → *D*-Val_6_ and *L*-Val_8_ → *L*-Val_12_. In general, the {^13^C, ^15^N, ^1^H} chemical shifts of the nuclei involved in hydrogen bonding (C_O_, N_amid_, H_amid_) reflect the strength of C_O_…H_amid_–N_amid_ contacts (see reference [24] for the most recent review of an influence of noncovalent interactions upon the SSNMR parameters). For these (C_O_, N_amid_, H_amid_) nuclei, values of their corresponding {^13^C, ^15^N, ^1^H} chemical shifts would be expected to rise with a decreasing length of the hydrogen bond (denoted as *R*; a value of *R* is given by the distance between C_O_ and N_amid_) if all other factors were equal (such an idealized dependence can be seen in Figure S1 that shows the ^15^N_amid_ data of the aforementioned NMA–DMF dimer). Of course, in a real system, there are many additional contributions influencing the chemical shift value. They stem from details of an angular dependence of the hydrogen bond; a presence of the secondary structural elements; a specific conformation of the backbone; and other effects. Nevertheless, for the triclinic polymorph, it follows from an inspection of predicted chemical shielding and a concomitant analysis of the geometry that the related chemical shifts increase with shortening of the hydrogen bond. For instance, in the molecule “B” *R* = 320 pm for *D*-Val_6_ → *D*-Val_2_, while the relevant {^13^C, ^15^N, ^1^H} chemical shifts are accordingly 175.9, 114, and 8.6 ppm. An analogous fragment in the molecule “A” has a significantly lower *R* of 300 pm, and higher {^13^C, ^15^N, ^1^H} chemical shifts amounting to 176.4, 123, and 9.2 ppm, respectively. This structural difference would thus result in a clear separation of the most downfield peak at 123 ppm from the rest of the signals in the ^15^N SSNMR spectrum (see Table 1). As a consequence, this peak could be utilized in distinguishing between the two crystallographically independent molecules in order to establish heteronuclear correlations for the full signal assignment of the triclinic polymorph.

### 2.3. The Monoclinic Polymorph

The *P*2_1_ structure of the monoclinic crystal form contains two symmetry-related molecules in the unit cell (visualized in the Graphical Abstract) together with one disordered *n*-octane molecule whose coordinates are not available [15]. Hence, only the corresponding uncomplexed VLM geometry was investigated here. It is available in the Supporting Materials file MONOCLINIC.PDB. Table 2 summarizes the predicted values of the SSNMR data and of the backbone dihedrals angles along with a specification of hydrogen bonding in this polymorph. Its packing and conformational features are analogous to both “A” and “B” molecules of the triclinic crystal structure described in the preceding paragraph. The monoclinic polymorph served to check the performance of the PW-PBE calculations for predictions of the ^13^C isotropic chemical shielding, σ^iso^, of α carbons in hydroxy acid residues, as their isotropic chemical shifts, δ^iso^, are available and were assigned to either *D*-Hyv or *L*-Lac by Kameda et al. (it is noted that one of the experimental peaks was affected by an occluded *n*-octane) [16]. They were fully assigned using the computed data (details are provided in Table S5). The model (see Section 2.2) σ^iso^ = −0.9452 × δ^iso^ + 165.2 ppm has an adjusted *R*^2^ value of 0.9008, standard deviation of 1.3 ppm, and a maximum deviation of 1.8 ppm. Importantly, the results reliably capture the dependence of the SSNMR data upon the solid-phase geometry of VLM, and their accuracy is at the level currently expected in NMR crystallography [25]. Thus, the PW-PBE calculations are applied with confidence to other nuclei and polymorphs examined in this work.

Based on a careful comparison of the notation used in references [14,15], a consistent numbering of the sites is adopted in Table 1 and Table 2. In the monoclinic polymorph, hydrogen bonds of the 1 → 4 type (sometimes they are used to describe “β-turn” motif in VLM structures, in analogy with the stabilized β-turns in proteins and polypeptides [26]) are *D*-Hyv_1_ → *L*-Val_4_, *L*-Lac_3_ → *D*-Val_6_, *L*-Lac_7_ → *D*-Val_10_, and *D*-Hyv_9_ → *L*-Val_12_. The 1 → 5 type hydrogen bonding is now *D*-Val_2_ → *D*-Val_10_ and *L*-Val_4_ → *L*-Val_8_. In spite of a fairly high degree of similarity between the VLM molecules of both polymorphs, values of the corresponding SSNMR parameters vary significantly (see Table 1 and Table 2) and reflect their sensitivity to seemingly small differences in the crystal packing and local effects. In particular, the hydrogen bond distance relevant for the *D*-Val_2_ → *D*-Val_10_ contact becomes *R* = 321 ppm and is almost the same as in the case of *D*-Val_6_ → *D*-Val_2_ in the “B” molecule of the triclinic polymorph (*R* = 320 pm as discussed in Section 2.2). However, due to a number of specific contributions to the chemical shifts of related (C_O_, N_amid_, H_amid_) nuclei, the {^13^C, ^15^N, ^1^H} values are now 172.7, 116, and 7.2 ppm, respectively, to be compared to 175.9, 114, and 8.6 ppm accordingly predicted for the triclinic polymorph.

### 2.4. The “Symmetric” Structural Motif

Due to the known tendency of VLM to polymorphism, two additional forms occurring in complexes were modeled, which should be helpful in their future studies. The XRD structure of a VLM complex with potassium cations, that is actually an adduct of VLM, KI_3_, and KI_5_ (see reference [17]), was considered. In the complex, VLM molecules are of a high (approximately *S*_6_) symmetry, and hence this polymorph is referred to as “symmetric” here. Its geometry was obtained by removing counterions and reoptimizing the unit cell parameters together with internal coordinates. The reoptimization resulted in only a small decrease of the unit-cell volume: formally, the unit cell shrank from 15.443 nm^3^ [17] to 15.134 nm^3^, but in fact, the corresponding primitive cell of the *C*222_1_ space group was used to make computations feasible, and the actual size was one-half of those values (further details are provided in Table S11). The resulting VLM geometry, included in the Supporting Materials as SYMMETRIC.PDB file, contains six hydrogen bonds of the 1 → 4 type. On the basis of a scrutiny of the backbone dihedral angles we suppose that the VLM ring is numbered consistently throughout Table 1, Table 2, Table 3 and Table 4 in order to aid in a comparison of the predicted SSNMR data. In the present case, the hydrogen bonds are *D*-Hyv_1_ → *L*-Val_4_, *L*-Lac_11_ → *D*-Val_2_, *L*-Lac_3_ → *D*-Val_6_, *D*-Hyv_5_ → *L*-Val_8_, *L*-Lac_7_ → *D*-Val_10_, and *D*-Hyv_9_ → *L*-Val_12_. It should be realized that despite a quasisymmetric shape of a VLM molecule in this conformation, there is a non-negligible variation in parameters of those six hydrogen bonds. Specifically, the values of *R* lie in an interval from 285.7 to 305.1 pm with a mean of 294.8 ppm and a standard deviation of this mean amounting to 7.9 pm. This variation is in turn manifested in differences between values of the {^13^C, ^15^N, ^1^H} chemical shifts of the (C_O_, N_amid_, H_amid_) nuclei involved in hydrogen bonding (see Table 3).

### 2.5. The “Propeller” Structural Motif

**Figure 3:**
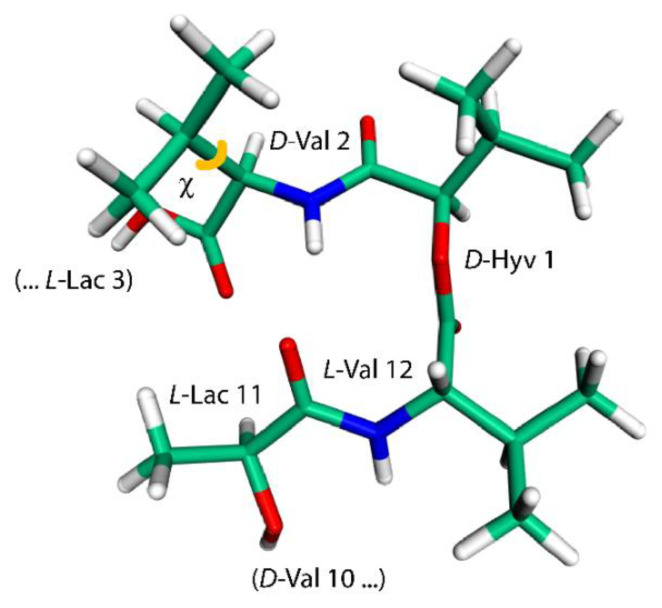
The model employed to calculate changes in the ^15^N chemical shielding of the amidic nitrogen of *D*-Val_2_ residue (this geometry features a χ, depicted in orange, of 163°).

The last intramolecular hydrogen-bonded conformation of VLM investigated here is the one found in its crystals containing also dimethyl sulfoxide (DMSO) molecules [18]. It resembles a shallow dish with isopropyl groups at the exterior. However, due to an approximate threefold rotation symmetry and three related bends, it can be likened to a three-blade propeller as well, and is referred to simply with “propeller” (see Figure 3 that shows one of the “blades” of such a “propeller”). This structure was investigated in an analogous way as the “symmetric” polymorph, namely, by removal of coordinates of solvent molecules from the available XRD structure and the reoptimization of the crystal unit-cell together with internal coordinates of VLM, which was followed by the prediction of the SSNMR parameters. Interestingly, a reduction of the unit-cell volume was small also in the case of the “propeller” polymorph, namely, the volume decreased from 7.897 to 7.642 nm^3^ (the resulting unit-cell parameters are provided in Table S11, while the VLM geometry in PROPELLER.PDB file). There are only three typical hydrogen bonds in this structure: *L*-Lac_11_ → *D*-Val_2_, *L*-Lac_3_ → *D*-Val_6_, and *L*-Lac_7_ → *D*-Val_10_, with *R* = 298, 301, and 312 pm, respectively. The hydrogen bonding affects the ^1^H chemical shifts of H_amid_ nuclei of amino acid residues in an expected way, namely, the values are much higher in *D*-valines (forming hydrogen bonds) than in *L*-valines (with amidic protons pointing in a direction where the nearest DMSO would be in the solvate structure [18], but where there is no acceptor in the present structure). However, the ^15^N chemical shifts of N_amid_ nuclei show an unanticipated pattern: the values are higher for *L*-valines than for hydrogen-bonded *D*-valines. An inspection of the ^15^N data in Table 1, Table 2, Table 3 and Table 4 reveals that those three *D*-valine nitrogens have in fact the lowest chemical shifts from among all N_amid_ sites investigated here. This uncommon situation is addressed in the next section by describing results of a simple model that includes an influence of isopropyl sidechains upon the ^15^N chemical shielding. In addition, the N_amid_–H_amid_ bond lengths, the eigenvalues and orientations of the ^15^N chemical shielding tensors, and the ^14^N isotropic quadrupolar interactions of valine residues in the “propeller” polymorph are discussed below. Also of note are the ^1^H chemical shifts of α protons of *L*-Lac residues in this structure. Their values are unusually high (in the 7 ppm range, see Table 4) and are discussed in terms of the C–H…O interactions.

## 3. Discussion

In the previous section, four solid-phase geometries of VLM together with corresponding sets of the {^13^C, ^15^N, ^1^H} chemical shifts of backbone nuclei are characterized by means of the PW DFT calculations with a main goal of describing the SSNMR markers of the respective polymorphs. It should also be mentioned that the known absence of intermolecular hydrogen bonds in the crystal packing of VLM is manifested in small values of dimerization energies of the nearest molecules. Those values are found to be similar to a *ΔE* of the model NMA–DMF system featuring just a single intramolecular hydrogen bond (see Section 2.1 for details). Due to the size of VLM dimers (1200 electrons), only a relatively small basis set (namely, TZVP, whose use resulted in an application of 4044 basis functions anyway) was employed in RIMP2 calculations, and their accuracy could not be assessed directly. However, it is worth the effort to compare the RIMP2/TZVP *ΔE* to an estimate of the corresponding complete basis-set limit (CBS) value of the aforementioned NMA–DMF dimer. The aug-cc-pV*X*Z family of basis sets was applied to compute the underlying RIMP2 energies, and the RIMP2/CBS *ΔE* was approximated using the mixed Gaussian/exponential form (see Materials and Methods). The resulting −32.9 kJ/mol is 122% of −26.9 kJ/mol obtained with the TZVP basis set. It is thus safe to assume that more than one half of a total dimerization energy of VLM was recovered by the RIMP2/TZVP calculations.

**Figure 4:**
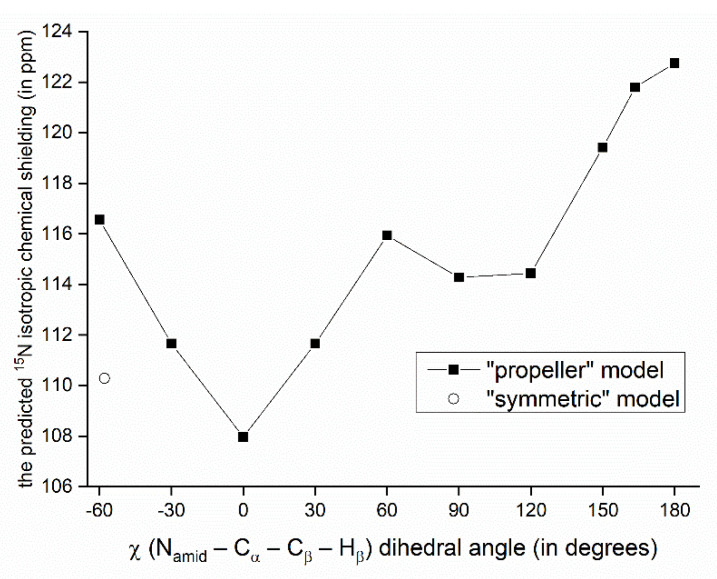
Variation of the GIAO-B3LYP/6-311++G(2d,2p) ^15^N chemical shielding with the sidechain dihedral angle. The point marked by an open circle has of a value of χ found in the PW PBE geometry of the “symmetric” polymorph.

The relationship between the SSNMR data of backbone nuclei and the solid-phase geometry of VLM conformations is explored in terms of parameters of the O…H_amid_–N_amid_ hydrogen bonding. Such an analysis is not straightforward due to several other contributions to the chemical shift of some specific nucleus. In particular, the ^15^N chemical shifts of N_amid_ nuclei of valine residues in the “propeller” structural motif do not follow the trend of an increase of the chemical shift value with weakening the hydrogen bond: the nitrogens of *D*-valines, which are hydrogen-bonded, are shielded more than those in *L*-valines. This shielding effect leads to unusually low values of the ^15^N chemical shift (predicted to be ca. 100 ppm which is to be compared to <113; 123> ppm interval of values in other polymorphs), and can be traced to an orientation of isopropyl sidechains with respect to amidic nitrogens of various structures. Specifically, in the case of *D*-valines in the “propeller” structure, a close contact occurs between their N_amid_ and one of C_γ_ carbons of the *D*-valine sidechain, and protons attached to that C_γ_. Since in other VLM structures the amidic nitrogens do not appear to come into such contact with neighboring sidechains, we assume that this interaction is mainly responsible for observed differences in the shielding. Our interpretation is supported by the model of a variation of the ^15^N chemical shielding with the sidechain dihedral angle, χ (see Figure 3 and Figure 4). This model was created from the PW PBE geometry of the “propeller” polymorph and was used to vary values of χ in an interval from −60° to +180°. For a total of 11 points from that interval, the ^15^N chemical shielding was obtained by the GIAO-B3LYP method (see Section 4 for details, and Table S8 for raw data) and plotted in Figure 4. It shows that N_amid_ sites become strongly shielded at high values of χ (which is the case of *D*-valines in the “propeller” structure, and is of course consistent with low values of their chemical shifts). It also shows that for a χ corresponding to *D*-Val_2_ in the “symmetric” polymorph, the ^15^N chemical shielding is relatively low. This is consistent with a high (122 ppm) ^15^N chemical shift value at that site, and corroborates our structural interpretation. Nevertheless, some other parameters related to hydrogen bonding in the “propeller” structural motif were also evaluated and are summarized in Table S9. As expected, [22], the N_amid_–H_amid_ bond lengths and the ^15^N chemical shielding anisotropies of N_amid_ nuclei are higher in *D*-valines (hydrogen-bonded residues) than in *L*-valines. The same trend holds for the ^14^N_amid_ isotropic quadrupolar shift [27], and also for an angle between the N_amid_–H_amid_ bond vector and an eigenvector associated with the least shielded component of the ^15^N_amid_ chemical shielding tensor. Values of that angle are higher by ca. 5° in *D*-valines as compared to *L*-valines (see Table S9). Moreover, since the ^17^O SSNMR can be usefully employed in studies of pharmaceutical compounds [28] and of hydrogen bonds [29], the related data for carbonyl oxygens are collected in Table S10.

**Figure 5:**
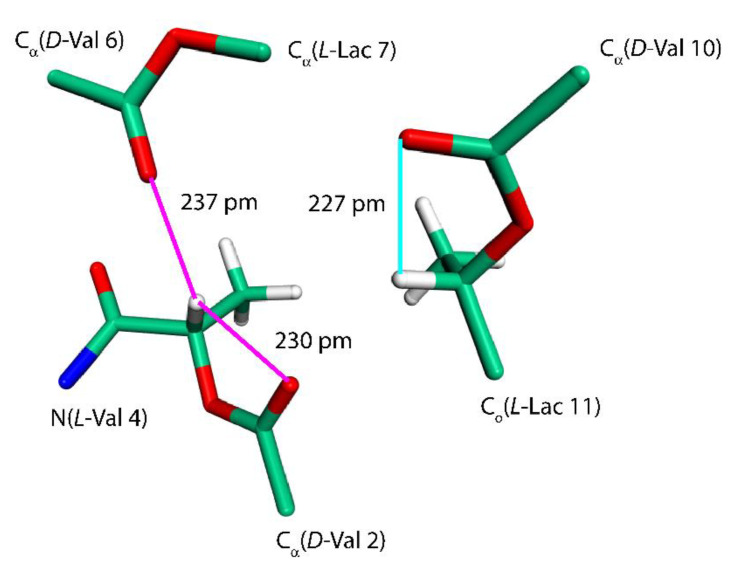
The fragment of a VLM molecule of the “propeller” polymorph. See the text for details.

Further insight into the values of some of the SSNMR parameters can be obtained by considering an effect of C–H…O hydrogen bonds [30]. We remark that the C–H…O contacts are generally weaker than typical hydrogen bonds (for instance, an NMA–DMF dimer featuring the hydrogen bond between the carbonyl oxygen of NMA and the formyl group of DMF has, in the PBE/aug-cc-pVQZ minimum, the RIMP2/CBS *ΔE* value of −26.5 kJ/mol that is to be compared to −32.9 kJ/mol discussed above). However, there are numerous sites at both amino and hydroxy acid residues of VLM acting as either an acceptor or a donor involved in the respective C–H…O interactions, which thus significantly contribute to the conformational stabilization. These interactions need to be taken into account in order to be able to explain strongly downfield shifts of *L*-Lac H_α_ nuclei in the propeller polymorph (see Table 4 and Figure 5). Interestingly, the H_α_ of *L*-Lac_3_ with the highest chemical shift value (7.3 ppm) participates in two C–H…O hydrogen bonds (marked by the magenta lines in Figure 5). Those hydrogen bonds are slightly shorter than a single C–H…O interaction involving H_α_ of *L*-Lac_11_ (the cyan line in Figure 5). In any case, these chemical shifts are expected to be an important SSNMR marker of this polymorph.

The strength of the SSNMR spectroscopy lies in its ability to specify the number of crystallographically independent molecules in the unit cell and thus easily distinguish between the monoclinic and triclinic polymorphs of VLM [16]. In this study, the NMR crystallography protocol has been applied to four VLM polymorphs in order to analyze the corresponding (^13^C/^15^N/^1^H) isotropic chemical shifts of (C_O_, N_amid_, H_amid_, H_α_) backbone nuclei in terms of structural features. Should isotope-enriched VLM samples become available, other SSNMR spectral markers might include the ^17^O parameters [31] or ^1^H_amid_–^15^N_amid_ and ^1^H_α_–^13^C_α_ dipolar couplings [32]. In addition, investigations of the chemical shift anisotropies could be performed [33,34,35] which would likely require measurements at very high (>20 T) magnetic fields [36,37,38].

## 4. Materials and Methods

The periodic PW DFT calculations were applied in the pseudopotential scheme [39,40,41] as implemented in the CASTEP 16.1 code [41]. Input files were prepared using the Materials Studio 2019 [42], and geometries were optimized with respect to the crystal-lattice energy approximated by the Perdew–Burke–Erzerhof (PBE) exchange-correlation functional [43]. In the case of computations that also optimized the crystal unit-cell parameters, the Tkatchenko–Scheffler dispersion-correction method was adopted [44]. The CASTEP calculations used the on-the-fly generated pseudopotentials and the settings consistent with “Fine” accuracy level of the Materials Studio software. Calculations were run on 60 cores of Intel^®^ Xeon^®^ Gold 6140 @3.70 GHz processors of the same high-performance server. We note that the optimization of the “propeller” polymorph converged exceedingly slowly: as many as 250 optimization cycles were needed to meet the convergence criteria, which took almost 18 days of CPU time of that server. For the resulting structures, the PW DFT chemical shielding tensors were predicted using the gauge-including projector augmented wave (GIPAW) method [45,46] combined with the PBE functional. The PW DFT electric-field gradient tensors were predicted, also using the PBE functional, by the method described in reference [47]. The resulting MAGRES files are obtainable.

The interaction energies were obtained from the supermolecular counterpoise-corrected calculations [48] of total energies, which were approximated by a sum of the Hartree–Fock energy and the correlation energy obtained using the resolution-of-the-identity integral approximation to the second-order Møller–Plesset energy (RIMP2) [49]. Standard triple-zeta valence plus polarization (TZVP) and augmented correlation-consistent polarized-valence basis sets (aug-cc-pV*X*Z, where *X* ∈ {D, T, Q} denotes the double-zeta (D), triple-zeta (T) and quadruple-zeta (Q) basis) were applied together with the corresponding auxiliary basis sets [50]. The RIMP2 calculations were carried out in Turbomole V7.1 [51]. The total energies were extrapolated to the complete basis set limit using an analytic solution (not shown) to the procedure described by equation 2 in reference [52].

The NMA–DMF dimers were fully optimized at the PBE/aug-cc-pVQZ level and verified to be minima of the potential energy surface by checking the values of harmonic vibrational frequencies. The chemical shielding tensors in model systems were predicted using the standard B3LYP combination of functionals applied with the standard 6-311++G(2d,2p) basis set, and with the GIAO [53,54] method to overcome the gauge problem. Additionally, for an NMA–DMF dimer the MP2-GIAO shielding tensors were predicted using the same basis set as in the B3LYP-GIAO calculations, in order to show that the chemical shielding changes are qualitatively the same (Tables S6 and S7, and Figure S1) [34]. The Gaussian 09 program package was used [55].

## Figures and Tables

**Table 1:** The PW PBE structural and spectral parameters of the triclinic crystalline phase of VLM (the values for the second crystallographically independent molecule are shown in parentheses).

Residue	H-Bonding Scheme	Corresponding {^13^C/^15^N/^1^H} NMR Chemical Shift (in ppm)					Dihedral Angle (in Degrees)	
		C_O_	C_α_	N_amid_	H_amid_	H_α_	φ	ψ
*D*-Hyv_1_	a ← d4 (a ← d4)	170.2 (171.0)	76.6 (75.1)	–	–	5.8 (6.2)	−2 (−7)	+94 (+102)
*D*-Val_2_	d → a11, a ← d6 (d → a11, a ← d6)	176.4 (175.9)	60.7 (58.5)	123 (114)	9.2 (8.6)	3.7 (4.8)	−124 (−135)	+65 (+64)
*L*-Lac_3_	none (none)	166.8 (166.3)	74.9 (75.6)	–	–	5.5 (5.2)	−11 (−11)	−68 (−68)
*L*-Val_4_	d → a1 (d → a1)	171.7 (172.3)	54.2 (53.8)	113 (112)	9.1 (8.3)	5.7 (5.9)	+68 (+76)	−109 (−107)
*D*-Hyv_5_	a ← d8 (a ← d8)	168.4 (169.6)	78.0 (78.3)	–	–	6.1 (6.3)	−6 (−11)	+153 (+142)
*D*-Val_6_	d → a2 (d → a2)	173.7 (173.7)	60.2 (61.1)	117 (116)	7.5 (8.0)	3.7 (3.7)	−130 (−129)	+57 (+62)
*L*-Lac_7_	a ← d10 (a ← d10)	171.8 (171.8)	70.3 (70.0)	–	–	6.1 (6.0)	+9 (+9)	−96 (−96)
*L*-Val_8_	d → a5, a ← d12 (d → a5, a ← d12)	177.9 (177.7)	61.5 (61.7)	120 (120)	9.3 (9.5)	4.0 (3.9)	+128 (+129)	−68 (−65)
*D*-Hyv_9_	none (none)	166.1 (166.3)	83.4 (83.2)	–	–	5.7 (5.6)	+3 (+4)	+82 (+81)
*D*-Val_10_	d → a7 (d → a7)	173.1 (173.3)	55.2 (54.6)	115 (116)	8.5 (8.6)	5.6 (5.9)	−66 (−66)	+106 (+104)
*L*-Lac_11_	a ← d2 (a ← d2)	171.3 (170.5)	72.9 (72.5)	–	–	6.7 (6.2)	+26 (+21)	−161 (−159)
*L*-Val_12_	d → a8 (d → a8)	173.7 (173.5)	60.8 (61.1)	116 (115)	8.5 (8.2)	4.0 (3.7)	+128 (+125)	−68 (−68)

**Table 2:** The PW PBE structural and spectral parameters of the monoclinic crystalline phase of VLM.

Residue	H-Bonding Scheme	Corresponding {^13^C/^15^N/^1^H} NMR Chemical Shift (in ppm)					Dihedral Angle (in Degrees)	
		C_O_	C_α_	N_amid_	H_amid_	H_α_	φ	ψ
*D*-Hyv_1_	a ← d4	168.9	76.2	–	–	6.3	−7	+142
*D*-Val_2_	d → a10	172.7	61.4	116	7.2	3.7	−130	+59
*L*-Lac_3_	a ← d6	171.5	71.1	–	–	6.1	+9	−96
*L*-Val_4_	d → a1, a ← d8	177.7	61.1	119	9.6	4.0	+127	−67
*D*-Hyv_5_	none	165.8	82.7	–	–	5.7	+2	+81
*D*-Val_6_	d → a3	172.7	54.8	115	8.5	5.6	−68	+106
*L*-Lac_7_	a ← d10	170.0	70.3	–	–	6.3	+22	−155
*L*-Val_8_	d → a4	173.2	61.5	115	7.9	3.6	+128	−67
*D*-Hyv_9_	a ← d12	171.2	74.2	–	–	6.0	−3	+95
*D*-Val_10_	d → a7, a ← d2	176.5	59.3	122	9.0	4.0	−125	+66
*L*-Lac_11_	none	166.9	73.6	–	–	5.5	−11	−77
*L*-Val_12_	d → a9	171.7	54.1	113	8.9	5.8	+74	−105

**Table 3:** The PW PBE structural and spectral parameters of the “symmetric” crystalline phase of VLM.

Residue	H-Bonding Scheme	Corresponding {^13^C/^15^N/^1^H} NMR Chemical Shift (in ppm)					Dihedral Angle (in Degrees)	
		C_O_	C_α_	N_amid_	H_amid_	H_α_	φ	ψ
*D*-Hyv_1_	a ← d4	170.3	75.9	–	–	5.8	+1	+101
*D*-Val_2_	d → a11	173.2	60.8	122	9.0	4.2	−123	+66
*L*-Lac_3_	a ← d6	172.5	72.3	–	–	5.4	−17	−79
*L*-Val_4_	d → a1	171.3	58.8	115	9.5	4.1	+107	−78
*D*-Hyv_5_	a ← d8	169.9	77.4	–	–	6.4	−8	+114
*D*-Val_6_	d → a3	169.7	59.0	121	9.0	4.3	−101	+83
*L*-Lac_7_	a ← d10	170.4	69.4	–	–	6.2	+8	−125
*L*-Val_8_	d → a5	170.8	59.2	119	9.8	4.3	+106	−80
*D*-Hyv_9_	a ← d12	170.3	75.5	–	–	6.2	+3	+115
*D*-Val_10_	d → a7	174.4	60.1	122	9.4	4.0	−128	+64
*L*-Lac_11_	a ← d2	171.3	71.8	–	–	5.2	−18	−76
*L*-Val_12_	d → a9	171.2	59.7	114	8.9	3.9	+118	−70

**Table 4:** The PW PBE structural and spectral parameters of the “propeller” crystalline phase of VLM.

Residue	H-Bonding Scheme	Corresponding {^13^C/^15^N/^1^H} NMR Chemical Shift (in ppm)					Dihedral Angle (in Degrees)	
		C_O_	C_α_	N_amid_	H_amid_	H_α_	φ	ψ
*D*-Hyv_1_	none	167.1	79.0	–	–	6.4	0	+104
*D*-Val_2_	d → a11	173.0	52.2	101	9.0	6.1	−177	+125
*L*-Lac_3_	a ← d6	171.9	70.9	–	–	7.3	+19	−146
*L*-Val_4_	none	173.5	59.9	113	7.2	4.6	+116	72
*D*-Hyv_5_	none	165.5	80.0	–	–	6.3	−4	+10
*D*-Val_6_	d → a3	174.5	53.7	100	8.5	6.5	−170	+121
*L*-Lac_7_	a ← d10	171.3	70.1	–	–	7.0	+35	−142
*L*-Val_8_	none	174.2	68.6	113	6.9	4.7	+122	−70
*D*-Hyv_9_	none	166.4	80.2	–	–	6.1	+3	+88
*D*-Val_10_	d → a7	172.5	53.4	99	8.3	6.0	−177	+121
*L*-Lac_11_	a ← d2	168.8	71.9	–	–	7.0	+25	−137
*L*-Val_12_	none	172.3	58.5	112	6.7	4.4	+110	−72

## Supplementary Materials

Supplementary materials can be found at https://www.mdpi.com/1422-0067/21/14/4907/s1. Tables S1–S4: the numbering of backbone atoms in the four polymorphs; Table S5: raw values of the SSNMR parameters of α carbons in the monoclinic and triclinic polymorphs; Tables S6 and S7: the ^15^N_amid_ chemical shielding data for various intermonomer separations in an NMA–DMF dimer; Figure S1: the distance dependence of the isotropic ^15^N_amid_ chemical shielding in an NMA–DMF dimer; Table S8: raw data used to create Figure 3; Tables S9 and S10: additional parameters describing the “propeller” polymorph; Table S11: the crystal unit-cell data of the four polymorphs.
