# Supplementary material for: Polymorphic Forms of Valinomycin Investigated by NMR Crystallography
Source: Int J Mol Sci. 2020 Jul 11;21(14):4907. doi: 10.3390/ijms21144907 (PMC7404035; doi:10.3390/ijms21144907)
Supplement: Supplementary file 1 [file ijms-21-04907-s001.zip › supplementary_files/SI.pdf]

Supplementary Information to "Polymorphic Forms of Valinomycin Investigated by NMR Crystallography" by Czernek & Brus (*Int. J. Mol. Sci.* **2020**)

Table S1. The numbering of atoms of the triclinic polymorph (whose structure is available in Supplementary Materials as TRICLINIC.PDB file) used to describe spectral and structural parameters discussed in the main text.

residue number	residue type	the backbone atom specification				
		X	C_o	C_α	*Y*	*Z*
1	*D*-Hyv	N57<br>(N223)	C21<br>(C188)	C20<br>(C187)	O67<br>(O234)	C7<br>(C175)
2	*D*-Val	O70<br>(O237)	C26<br>(C193)	C25<br>(C192)	N57<br>(N223)	C21<br>(C188)
3	*L*-Lac	N58<br>(N224)	C31<br>(C197)	C30<br>(C196)	O70<br>(O237)	C26<br>(C193)
4	*L*-Val	O73<br>(O240)	C34<br>(C200)	C33<br>(C199)	N58<br>(N224)	C31<br>(C197)
5	*D*-Hyv	N59<br>(N225)	C39<br>(C205)	C38<br>(C204)	O73<br>(O240)	C34<br>(C200)
6	*D*-Val	O76<br>(O243)	C43<br>(C209)	C42<br>(C208)	N59<br>(N225)	C39<br>(C205)
7	*L*-Lac	N60<br>(N226)	C48<br>(C214)	C47<br>(C213)	O76<br>(O243)	C43<br>(C209)
8	*L*-Val	O62<br>(O229)	C51<br>(C217)	C50<br>(C216)	N60<br>(N226)	C48<br>(C214)
9	*D*-Hyv	N56<br>(N222)	C3<br>(C171)	C2<br>(C170)	O62<br>(O229)	C51<br>(C217)
10	*D*-Val	O65<br>(O232)	C9<br>(C177)	C8<br>(C176)	N56<br>(N222)	C3<br>(C171)
11	*L*-Lac	N55<br>(N221)	C16<br>(C183)	C15<br>(C181)	O65<br>(O232)	C9<br>(C177)
12	*L*-Val	O67<br>(O234)	C7<br>(C175)	C1<br>(C169)	N55<br>(N221)	C16<br>(C183)

Table S2. The numbering of atoms of the monoclinic polymorph (whose structure is available in Supplementary Materials as MONOCLINIC.PDB file) used to describe spectral and structural parameters discussed in the main text.

residue number	residue type	the backbone atom specification				
		X	C_o	C_α	*Y*	*Z*
1	*D*-Hyv	N20	C34	C30	O2	C29
2	*D*-Val	O5	C39	C35	N20	C34
3	*L*-Lac	N21	C42	C40	O5	C39
4	*L*-Val	O8	C47	C43	N21	C42
5	*D*-Hyv	N22	C52	C48	O8	C47
6	*D*-Val	O11	C57	C53	N22	C52
7	*L*-Lac	N23	C60	C58	O11	C57
8	*L*-Val	O14	C65	C61	N23	C60
9	*D*-Hyv	N24	C70	C66	O14	C65
10	*D*-Val	O17	C75	C71	N24	C70
11	*L*-Lac	N19	C78	C76	O17	C75
12	*L*-Val	O2	C29	C25	N19	C78

Table S3. The numbering of atoms of the 'symmetric' polymorph (whose structure is available in Supplementary Materials as SYMMETRIC.PDB file) used to describe spectral and structural parameters discussed in the main text.

residue number	residue type	the backbone atom specification				
		X	C_o	C_α	*Y*	*Z*
1	*D*-Hyv	N146	C4	C1	O151	C52
2	*D*-Val	O167	C41	C23	N146	C4
3	*L*-Lac	N149	C47	C44	O167	C41
4	*L*-Val	O152	C53	C50	N149	C47
5	*D*-Hyv	N147	C5	C2	O152	C53
6	*D*-Val	O168	C42	C29	N147	C5
7	*L*-Lac	N150	C48	C45	O168	C42
8	*L*-Val	O153	C54	C51	N150	C48
9	*D*-Hyv	N145	C6	C3	O153	C54
10	*D*-Val	O166	C40	C22	N145	C6
11	*L*-Lac	N148	C46	C43	O166	C40
12	*L*-Val	O151	C52	C49	N148	C46

Table S4. The numbering of atoms of the ‘propeller’ polymorph (whose structure is available in Supplementary Materials as PROPELLER.PDB file) used to describe spectral and structural parameters discussed in the main text and shown also in Table S9.

residue number	residue type	the backbone atom specification					
		X	C_o	C_α	*Y*	*Z*	H_{amid}
1	*D*-Hyv	N15	C10	C9	O8	C3	–
2	*D*-Val	O22	C17	C16	N15	C10	H96
3	*L*-Lac	N27	C24	C23	O22	C17	–
4	*L*-Val	O34	C29	C28	N27	C24	H109
5	*D*-Hyv	N41	C36	C35	O34	C29	–
6	*D*-Val	O48	C43	C42	N41	C36	H126
7	*L*-Lac	N53	C50	C49	O48	C43	–
8	*L*-Val	O60	C55	C54	N53	C50	H139
9	*D*-Hyv	N67	C62	C61	O60	C55	–
10	*D*-Val	O74	C69	C68	N67	C62	H156
11	*L*-Lac	N1	C77	C75	O74	C69	–
12	*L*-Val	O8	C3	C2	N1	C77	H79

Table S5. The specification and values (in ppm) of the ^{13}C SSNMR parameters of α carbons in the monoclinic and triclinic polymorphs.

polymorph							
triclinic				monoclinic			
experimental		PW-PBE		experimental		PW-PBE	
peak	*ii* component: δ_{ii}	site	*ii* component: σ_{ii}	peak of	δ^{iso}	site	σ^{iso}
H1	11: 100	*D*-Hyv 9	11: (58.6682+59.6540)/2	D-Hyv	81.3	D-Hyv 5	86.8263
H1	22: 78	*D*-Hyv 9	22: (93.5526+93.1414)/2				
H1	33: 66	*D*-Hyv 9	33: (106.1981+106.1069)/2				
H2	11: 105	*D*-Hyv 5	11: (58.5057+53.6124)/2	D-Hyv	77.1	D-Hyv 1	93.3301
H2	22: 68	*D*-Hyv 5	22: (102.1205+106.2545)/2				
H2	33: 59	*D*-Hyv 5	33: (113.7510+113.7419)/2				
H3	11: 96	*D*-Hyv 1	11: (67.5024+65.9476)/2	D-Hyv	75.9	D-Hyv 9	95.2835
H3	22: 73	*D*-Hyv 1	22: (98.2170+102.3314)/2				
H3	33: 58	*D*-Hyv 1	33: (113.1168+114.8188)/2				
L1	11: 97	*L*-Lac 3	11: (64.1973+66.8905)/2	L-Lac	72.9	*L*-Lac 11	95.8564
L1	22: 80	*L*-Lac 3	22: (89.5981+86.6424)/2				
L1	33: 45	*L*-Lac 3	33: (129.8963+128.1672)/2				
L2	11: 102	*L*-Lac 11	11: (59.5495+59.5399)/2	L-Lac	71.0	*L*-Lac 3	98.3721
L2	22: 68	*L*-Lac 11	22: (100.0+101.0135)/2				
L2	33: 43	*L*-Lac 11	33: (130.1504+130.6031)/2				
L3	11: 98	*L*-Lac 7	11: (63.9822+63.8139)/2	L-Lac	68.8	*L*-Lac 7	99.2024
L3	22: 71	*L*-Lac 7	22: (99.8615+100.3297)/2				
L3	33: 38	*L*-Lac 7	33: (133.6399+134.2346)/2				

Table S6. The values (in ppm) of principal components of the ^{15}N chemical shielding tensor of N_{amid}, provided by the GIAO-MP2 and GIAO-B3LYP methods applied with the 6-311++G(2d,2p) basis set, for various separations (in pm) of the *N*-methylacetamide – dimethylformamide dimer whose equilibrium structure (marked with '*') was obtained by the RIMP2/aug-cc-pVTZ geometry optimization carried out in a routine way using the RICC2 module of TURBOMOLE V7.1 (http://www.cosmologic.de/turbomole/home.html); *R* is the distance between the oxygen in dimethylformamide and the nitrogen in *N*-methylacetamide.

R (rounded)	MP2			B3LYP		
	σ_{11}	σ_{22}	σ_{33}	σ_{11}	σ_{22}	σ_{33}
250.7	50.9569	186.2322	234.2069	16.7613	169.0305	208.7232
260.7	53.2847	183.9515	238.2803	19.3408	166.4098	212.9477
270.7	56.8237	180.0219	243.7221	23.2693	161.8629	218.5301
280.7	57.528	179.1745	244.6701	24.0542	160.8806	219.4882
290.7*	58.1727	178.3815	245.4908	24.7743	159.9613	220.3115
300.7	58.7639	177.6403	246.2029	25.4359	159.1021	221.0196
310.7	59.3067	176.9483	246.8225	26.0444	158.3003	221.6296
320.7	61.0703	174.6403	248.5804	28.0279	155.6391	223.3229
340.7	62.3433	172.9421	249.5836	29.4673	153.7033	224.2835
370.7	63.6631	171.2098	250.3568	30.9504	151.7572	224.9977
400.7	64.5446	170.1236	250.7250	31.9371	150.5436	225.2851

Table S7. The values (in ppm) of the isotropic chemical shielding, σ^{iso}, and of the chemical shielding anisotropy, CSA_a, of the ^{15}N sites described in the caption to Table S6.

R (rounded)	MP2		B3LYP	
	σ^{iso}	CSA_a	σ^{iso}	CSA_a
250.7	157.1320	164.5927	131.5050	175.5147
260.7	158.5055	164.6949	132.8994	175.0410
270.7	160.1892	164.5699	134.5541	173.9916
280.7	160.4575	164.4836	134.8077	173.7106
290.7*	160.6817	164.3805	135.0157	173.4250
300.7	160.8690	164.2647	135.1859	173.1378
310.7	161.0258	164.1404	135.3248	172.8522
320.7	161.4303	163.5930	135.6633	171.7642
340.7	161.6230	163.0413	135.8180	170.8354
370.7	161.7432	162.3038	135.9018	169.7245
400.7	161.7977	161.7199	135.9219	168.8746

$\sigma^{iso} = \frac{1}{3}(\sigma_{11} + \sigma_{22} + \sigma_{33})$; $CSA_a = \sqrt{\sigma_{11}^2 + \sigma_{22}^2 + \sigma_{33}^2 + \sigma_{11}\sigma_{22} + \sigma_{22}\sigma_{33} + \sigma_{33}\sigma_{11}}$

Figure S1. The distance dependence of the ^{15}N σ^{iso} data (in ppm) described in Tables S6 and S7.

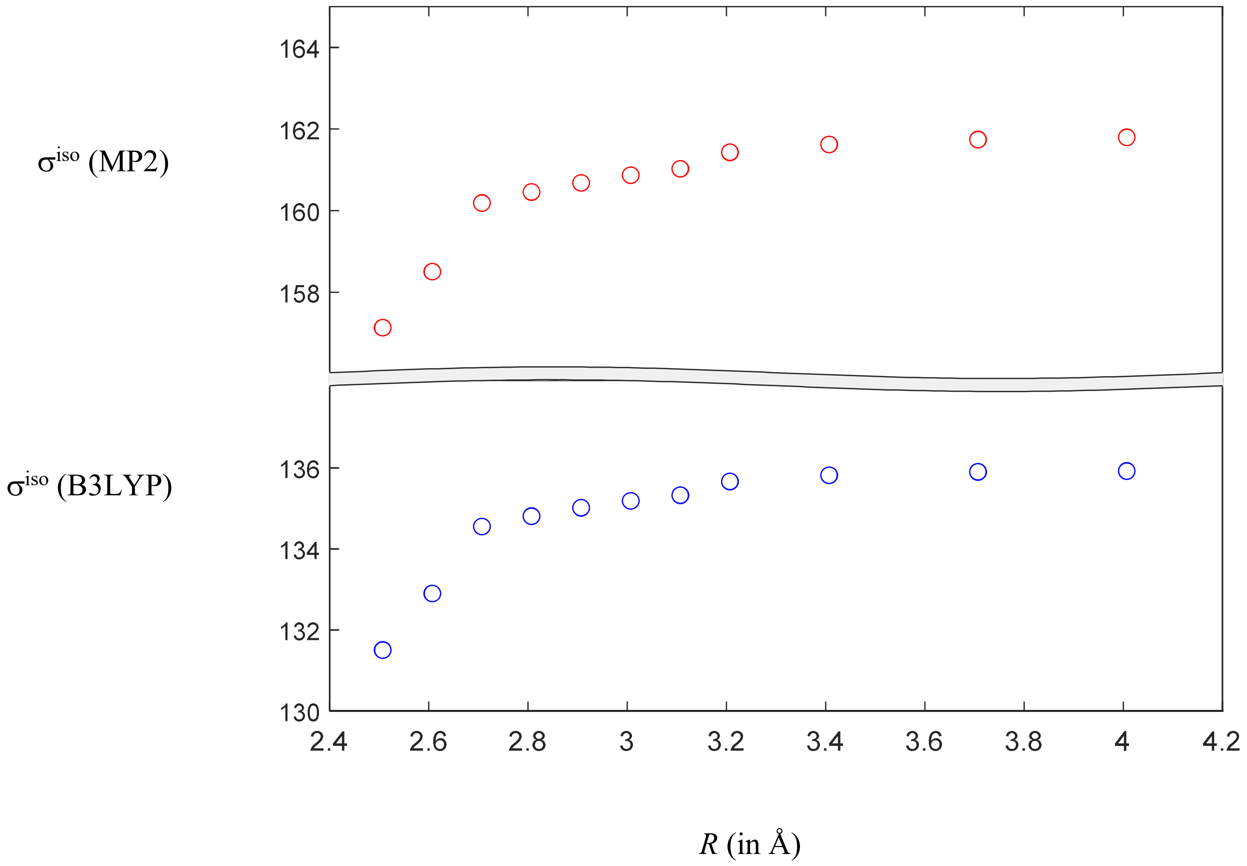

Figure S1 was created employing the 'Break Y Axis' script; citation: MikeCF (2020). Break Y Axis (https://www.mathworks.com/matlabcentral/fileexchange/45760-break-y-axis), MATLAB Central File Exchange. Retrieved May 18, 2020.

Table S8. Actual values of the data plotted in Figure 2 of the main text.

χ (deg.)	σ^{iso} (ppm)	comment
180	122.7514	unrelaxed
163.43	121.7925	taken directly from the PW-PBE structure of the 'propeller' polymorph
150	119.4218	unrelaxed
120	114.4452	unrelaxed
90	114.2811	unrelaxed
60	115.9387	unrelaxed
30	111.6539	unrelaxed
0	107.9671	unrelaxed
-30	111.6577	unrelaxed
-60	116.5615	unrelaxed
-57.84	110.2896	taken directly from the PW-PBE structure of the 'symmetric' polymorph

Table S9. Selected structural and spectral parameters of the amidic nitrogens of the ‘propeller’ polymorph. The N_{amid}–H_{amid} distances (rNH) are in picometers, all angles are in degrees, and {ε_{11}, ε_{22}, ε_{33}, CSA_a, ν_Q} data are in ppm.

residue number	residue type	r_{NH}	α	β	γ	ε_{11}	ε_{22}	ε_{33}	CSA_a	ν_Q
2	*D*-Val	102.52	2.2	19.8	34.4	41.7	52.1	210.3	163.6	406
4	*L*-Val	101.64	5.4	15.0	11.5	43.3	78.5	217.2	159.3	369
6	*D*-Val	102.34	0.7	19.9	37.0	39.8	51.6	209.5	164.1	391
8	*L*-Val	101.58	1.6	16.0	8.0	39.7	80.1	218.0	162.0	370
10	*D*-Val	102.28	5.0	20.3	34.7	37.2	50.9	208.0	164.3	417
12	*L*-Val	101.50	0.6	14.7	8.0	44.2	78.6	212.8	154.3	389

The {α, β, γ} angles describe an orientation of the ^{15}N chemical shielding tensor (CST) in the peptide plane. The reference plane is defined by the respective N_{amid}; C_o; C_α atoms (the numbering is provided in Table S4). The angle α is defined by a projection onto this plane of the eigenvector, ξ_1, associated with the smallest eigenvalue of given CST. The angle β is subtended between a ξ_1 and the related N_{amid}–H_{amid} bond vector (the corresponding H_{amid} atom numbers are collected in Table S4). The angle γ is defined by an angle between the normal to N_{amid}; C_o; C_α plane and an eigenvector associated with the mid-shielded eigenvalue of the ^{15}N CST in question.

The {ε_{11}, ε_{22}, ε_{33}} values are estimates of ^{15}N chemical shift tensor components (see the main text). The CSA_a values are estimates of the chemical shift anisotropy contribution to autocorrelation:

$$\mathrm{CSA_a} = \sqrt{\varepsilon_{11}^2 + \varepsilon_{22}^2 + \varepsilon_{33}^2 + \varepsilon_{11}\varepsilon_{22} + \varepsilon_{22}\varepsilon_{33} + \varepsilon_{33}\varepsilon_{11}}$$

The ν_Q values are estimates of the ^{14}N isotropic quadrupolar shift at the Larmor frequency, ν_0, of 54.207 MHz (i.e., at a spectrometer with the magnetic field strength of 17.6 T):

$$\upsilon_Q = \frac{3}{40}\frac{C_Q^2}{\upsilon_0^2}\left(1 + \frac{1}{3}\eta_Q^2\right)$$

where C_Q is the ^{14}N quadrupolar coupling constant and η_Q is the asymmetry parameter of the corresponding ^{14}N electric field-gradient tensor (both parameters were obtained from a PW-PBE calculation and are not shown).

Table S10. The raw values of the ^{17}O solid-state NMR parameters predicted for the carbonyl oxygens of the 'propeller' polymorph. The {σ_{11}, σ_{22}, σ_{33}, σ^{iso}} chemical shielding data are in ppm, the quadrupolar coupling constant, C_Q, are in MHz, and the asymmetry parameters, η_Q, are unitless. The 'atom name' entry refers to the Supplementary Materials PROPELLER.PDB file.

residue number	residue type	atom name	σ_{11}	σ_{22}	σ_{33}	σ^{iso}	C_Q	η_Q
1	*D*-Hyv	O4	-309.2431	-164.6439	348.5169	-41.7901	8.4176	0.2655
2	*D*-Val	O6	-375.8535	-233.1605	303.7083	-101.7686	8.9496	0.1699
3	*L*-Lac	O8	-352.8890	-179.2573	329.1473	-67.6663	9.0567	0.2195
4	*L*-Val	O9	-307.5522	-233.0764	308.3946	-77.4113	8.6437	0.0952
5	*D*-Hyv	O10	-321.6722	-176.4025	351.7048	-48.7900	8.5646	0.2124
6	*D*-Val	O12	-352.9269	-215.7830	307.6527	-87.0191	8.7892	0.1207
7	*L*-Lac	O13	-354.3076	-175.3749	332.6280	-65.6848	9.0391	0.2042
8	*L*-Val	O15	-332.1441	-250.5885	308.7396	-91.3310	8.7529	0.1381
9	*D*-Hyv	O16	-321.7959	-180.1362	354.8578	-49.0248	8.5511	0.1964
10	*D*-Val	O18	-370.5943	-229.2862	305.3186	-98.1873	8.9084	0.1493
11	*L*-Lac	O19	-351.1187	-179.7139	330.6891	-66.7145	9.0047	0.2224
12	*L*-Val	O21	-312.4340	-234.2772	314.1272	-77.5280	8.6544	0.1201

Table S11. The solid phase structures investigated in this work (the unit cell parameters are shown which were actually used in analyses described in the main text).

polymorph	source [1]	space group	*a* [Å]	*b* [Å]	*c* [Å]	*α* [°]	*β* [°]	*γ* [°]	*V* [$Å^3$]
triclinic	VALINO	*P* 1 (#1)	22.285	10.360	14.525	90.06	105.26	93.31	3229
monoclinic	VALINM30	$P\,2_1$ (#4)	23.144	10.347	14.526	90	99.57	90	3430
"symmetric"	VALINK [2]	$C\,222_1$ (#20) [3]	14.274	14.274	44.497	90	90	56.58	7567
"propeller"	GEYHOH [4]	$P\,2_12_12_1$ (#19)	16.618	24.924	18.451	90	90	90	7642

Footnotes to Table S11:

[1] The Cambridge Crystallographic Database identifier (https://www.ccdc.cam.ac.uk/).

[2] Counterions removed before the PW DFT optimization.

[3] The PW DFT optimization carried out in the corresponding primitive cell.

[4] The solvate atoms removed before the PW DFT optimization.
